# A comparative study of the vibro-impact capsule systems with one-sided and two-sided constraints

**DOI:** 10.1007/s11071-017-3500-7

**Published:** 2017-04-07

**Authors:** Yao Yan, Yang Liu, Maolin Liao

**Affiliations:** 1 School of Aeronautics and Astronautics, University of Electronic Science and Technology of China, Chengdu, 611731 China; 2 College of Engineering Mathematics and Physical Sciences, University of Exeter, Rennes Drive, Exeter, EX4 4RN UK; 3 CNPC Drilling Research Institute, Beijing, 102206 China

**Keywords:** Vibro-impact, Non-smooth dynamical system, Stick-slip, Optimisation, Energy consumption

## Abstract

This paper studies the dynamics of the vibro-impact capsule systems with one-sided and two-sided soft constraints under variations of various system and control parameters, including mass ratio, stiffness ratio, gap of contact, and amplitude and frequency of external excitation. The aim of this study is to optimise the progression speed and energy consumption of the capsule and minimise the required cabin length for prototype design used for engineering pipeline inspection. Our studies focus on three systems: the capsule with a right constraint, the capsule with a right and a weak left constraints, and the capsule with a right and a strong left constraints. Bifurcation analyses show that the behaviour of the capsule with one-sided constraint is mainly periodic, and the dynamic responses of the other two capsules with two-sided constraints become complex when the stiffness of the left constraint increases. Based on our extensive comparisons, the following optimisation strategies are recommended. When the capsule speed is paramount, one can employ the two-sided capsule with a weak left constraint under large amplitude of excitation. When energy consumption is taken into account, the one-sided capsule is preferable. When a miniaturized prototype is needed, the two-sided capsule with a strong left constraint is the best choice.

## Introduction

Pipelines play an important role in a large number of modern industries [1, 2], transporting fluids from one location to another, whether from one side of a factory to the other or across the breadth of entire continents. They are essential assets within water supplies, oil and gas production, and many other vital industries throughout the globe. Pipelines vary greatly in diameter, length, construction material, and location. With many pipelines being located in remote and harsh locations, such as being located underground or running along the seabed, access for inspection, maintenance and repair work could be extremely difficult. The challenges also include accurately locating leaks or blockages within operational pipelines, and monitoring corrosion or deterioration on internal surfaces. It becomes a particularly costly issue if the pipeline has to be drained and production stopped while repair work takes place. Therefore, pipeline inspection devices capable of moving independently, with or against product flow would yield significant advantages over traditional pressure driven inspection tool [3] in certain situations. In recent years, investigation of the self-propelled mechanism moving rectilinearly under internal vibration force when overcoming medium resistance has attracted great attention from researchers, e.g. [4–8]. The principle of such mechanism is that the rectilinear motion can be obtained by overcoming external resistance described as dry friction using an additional internal mass interacting with the main body of the system. The ability to move independently without any external moving parts makes it ideally suited to move in harsh and complex environments, where external moving parts may either pose a hazard to the surroundings or where they would be likely to be broken, corroded, or blocked up by the working environment.

This paper studies the optimisation of the vibro-impact capsule systems [8–10] with one-sided and two-sided amplitude constraints for engineering pipeline inspection in terms of its average speed of progression, physical dimension, and energy consumption. Optimisation of motion of the self-propelled mechanism regarding to average speed of progression has been an active subject of scientific research. For example, Chernousko [4] pioneeringly studied the optimum rectilinear motion of a two-mass system and obtained the optimum control parameters for the maximum mean velocity of the system. In [6, 11], optimisation of a mobile system with an internal acceleration-controlled mass was considered to obtain the maximum forward mean speed. Later on, Fang and Xu [12] studied the dynamics of a multibody system consisting of three modules of such vibration-driven mobile system. Considering the controlled motion a rigid body in the horizontal plane, Zhan and Xu [13] used three internal acceleration-controlled masses to drive the system. Recently, optimisation of two-dimensional motion of the vibration-driven system has been extensively studied, e.g. [14, 15]. However, these studies focused on theoretical calculation of the maximum mean speed, and few works concerned optimisation of the mobile system from a practical design point of view, such as physical dimension and energy consumption. Vetchanin et al. [16] investigated the characteristics of motion of a rigid body with variable internal mass distribution in a viscous fluid, showing the possibility of self-propulsion of the body in an arbitrary given direction. In [17], experimental verification of the vibro-impact capsule system was carried out. The conducted bifurcation analyses indicated that a fine tuning of the control parameters, such as the stiffness of the support spring, and the frequency and the amplitude of excitation, can significantly improve the average rate of progression. In [18], optimisation of the vibro-impact capsule system for the best progression in fluid environment was studied, focusing on the choice of the excitation parameters and the shape of the capsule. This paper will further study the optimisation of the vibro-impact capsule system regarding to its physical dimension and energy consumption through bifurcation analysis. Three capsule dynamics with one-sided and two-sided amplitude constraints will be compared in order to obtain the best design parameters for prototyping.

**Fig. 1:**
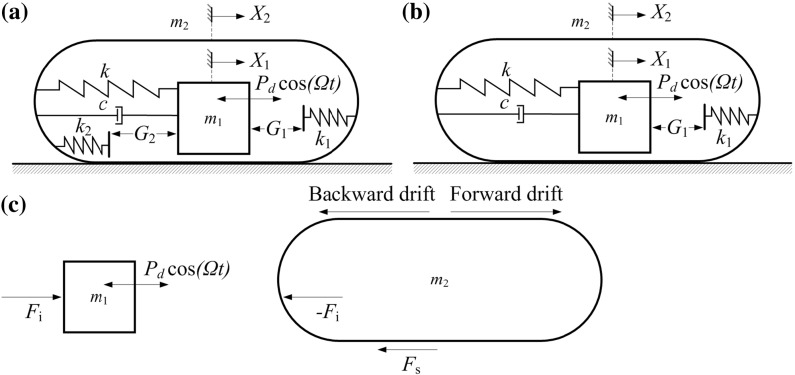
Physical models of the vibro-impact capsule systems, with **a** double-sided constraints, and **b** one-sided constraint. **c** Free body diagrams of the inner mass $m_1$ and the capsule $m_2$

The dynamics of the vibro-impact capsule system, which consists of a capsule main body interacting with a harmonically driven internal mass, has been studied extensively by Liu et al. [8–10, 17–20]. In [8], the model of the vibro-impact capsule system was firstly studied to provide a fundamental understanding of its dynamics. Dynamics of the system in various frictional environments was investigated in [9], and numerical results show that the behaviour of the system becomes very complex when the capsule is moving in fluid, but the nature of the friction mechanism becomes less significant when the weight of the internal mass is smaller than the weight of the capsule. In [10], nonlinear dynamics analysis has been conducted to identify the optimal amplitude and frequency of the applied force to achieve the required motion and the maximal speed. In [19], Páez Chávez et al. studied two practical problems for the capsule system, which were maximising the rate of progression and directional control of the system by following a typical period-1 trajectory by means of path-following techniques. However, the above studies were based on the dynamics of the capsule system with one-sided constraint, and the performance of the system with two-sided constraints has not been investigated. Thus, the contribution of this paper is to study the capsule system with two-sided soft constraints and understand how does the additional constraint affect the performance of the capsule. There are some existing studies on the vibro-impact systems with two-sided constraints, but most of them focused on the systems with bilateral rigid constraints. For example, Gutiérrez and Arrowsmith [21] considered a representative model of the doubly constrained impacting system, and studied the control strategies for preservation and annihilation of experimental and analytical resonant periodic orbits. Lee and Yan [22] developed a position control method for the impact oscillator with asymmetrical double-sided endstops, which can keep the stable and the unstable oscillators in a desired position. Luo et al. [23] studied the vibro-impact dynamics of a two-degree-of-freedom periodically forced system with a bilateral clearance. Response analysis for a vibro-impact Duffing system with bilateral barriers under external and parametric Gaussian white noises was carried out by Yang et al. [24]. Very recently, Kumar et al. [25] analysed a stochastically excited vibro-impact Duffing–Van der Pol oscillator with bilateral rigid barriers. However, the literature on the vibro-impact systems with double-sided soft constraints is rather limited. Andreaus and Angelis [26] studied the dynamic response of a single-degree-of-freedom oscillator constrained by two unilateral nonlinear bumpers. Hao et al. [27] developed a two-sided damping constraint control strategy for the quasi-zero stiffness isolator. The study by Ing [28] revealed that, in a near symmetrical system, the degree of asymmetry was found to have a vast effect on the response of a double-sided constraint impact oscillator. In this paper, we will investigate whether such asymmetry can be used for optimising the physical performance of the capsule system, e.g. progression rate, power efficiency, and capsule dimension. In practice, the stroke length of the internal mass, i.e. the maximum travel distance of the mass in one period of motion, constrains the physical dimension and power consumption of the capsule system, which in turn affects the performance of the system. Thus, it is reasonable to carry out a comparative study in this paper, which can provide a better insight for the design of such system with consideration of its physical performance, and this is the novelty of this paper.

The rest of this paper is organised as follows. In Sect. 2, mathematical modelling of the vibro-impact capsule systems with one-sided and two-sided constraints is studied. In Sect. 3, bifurcation analysis of the capsule system is conducted through varying various system and control parameters. Optimisation of energy consumption and cabin length are considered in Sect. 4, and finally, some concluding remarks are drawn in Sect. 5.

## Mathematical modelling

### Description of the capsule system

Consider a two degrees-of-freedom system as shown in Fig. 1a, which is composed of a movable internal mass $m_1$ interacting with a rigid capsule $m_2$ via a primary linear spring with stiffness *k* and a viscous damper with damping coefficient *c*. The internal mass is driven by an external harmonic force with amplitude $P_d$ and frequency $\Omega$. In practice, this can be implemented using a linear actuator (e.g. solenoid [29]). It is worth noting that the interaction between the rod ($m_1$) and the main body ($m_2$) of the actuator is approximated using a linear spring and a viscous damper in this paper, and the verification of such assumption was carried out in [17]. On the right of the internal mass, a weightless plate is connected to the capsule by a linear spring with stiffness $k_1$, and a secondary weightless plate is connected to the capsule by a linear spring with stiffness $k_2$ on the left of the internal mass. Here, $X_1$ and $X_2$ represent the absolute displacements of the internal mass and the capsule, respectively. The internal mass will contact with the right plate when the relative displacement $X_1-X_2$ is larger or equals to the gap $G_1$, or contact with the left plate when the relative displacement $X_2-X_1$ is larger or equals to the gap $G_2$. When the left spring is removed (i.e. $k_2=0$) as shown in Fig. 1b, the system has only one-sided constraint which has been thoroughly studied by Liu et al. [8–10].

### Equations of motion

Due to the non-smoothness introduced by the gaps and the friction, equations of motion of the capsule system should be considered in different phases. For the non-smoothness of gaps $G_1$ and $G_2$, mutual interaction between $m_1$ and $m_2$ has three different cases: no contact ($X_1-G_1-X_2<0$ and $X_2-G_2-X_1<0$), right contact ($X_1-G_1-X_2\ge 0$) and left contact ($X_2-G_2-X_1\ge 0$). Thus, the interactive force is given as

1 $$\begin{aligned} F_i= {\left\{ \begin{array}{ll} -c(\dot{X}_1-\dot{X}_2)-k(X_1-X_2),&{}\text {(no contact)}\\ -c(\dot{X}_1-\dot{X}_2)-k(X_1-X_2)-k_1(X_1-G_1-X_2),&{}\text {(right contact)}\\ -c(\dot{X}_1-\dot{X}_2)-k(X_1-X_2)-k_2(X_1+G_2-X_2),&{}\text {(left contact)} \end{array}\right. } \end{aligned}$$

or written as

2 $$\begin{aligned} F_i= & {} -c(\dot{X}_1-\dot{X}_2)-k(X_1-X_2)\nonumber \\&-H_{1}k_1(X_1-G_1-X_2)\nonumber \\&-H_{2}k_2(X_1+G_2-X_2), \end{aligned}$$

where $H_1$ and $H_2$ are the Heaviside functions given by

3 $$\begin{aligned} \begin{aligned} H_1=\,&H(X_1-G_1-X_2),\\ H_2=\,&H(X_2-G_2-X_1). \end{aligned} \end{aligned}$$

The second non-smoothness of the system is introduced by the friction $F_s$ between the capsule and its supporting surface when the capsule moves horizontally ($\dot{X}_2\ne 0$) as depicted in Fig. 1c. Here, the Coulomb friction model is used to describe the frictional force given by

4 $$\begin{aligned} F_s=-\text {sign}(\dot{X}_2)P_f, \end{aligned}$$

where $P_f=\mu (m_1+m_2)g, \mu$ is the friction coefficient between the capsule and the environmental surface, and *g* is the acceleration due to gravity. When the capsule is stationary ($\dot{X}_2=0$), two situations could happen. If the elastic force acting on the capsule is greater than the threshold of friction, i.e. $\left| F_i\right| >P_f$, the capsule begins to move and the direction of friction force is opposite to the elastic force. At this moment, the dry friction force is calculated as

5 $$\begin{aligned} F_s=-\text {sign}(F_i)P_f, \end{aligned}$$

When the force acting on the capsule from the internal mass is smaller than the threshold of friction, i.e. $\left| F_i\right| \le P_f$, the friction force becomes static which is calculated as

6 $$\begin{aligned} F_s=-F_i. \end{aligned}$$

Considering all the conditions above, the comprehensive friction force can be written as

7 $$\begin{aligned} F_s= & {} -(1-\Delta _\mathrm{v})S_{v}P_{f}-\Delta _\mathrm{v}H_{f}S_{f}P_{f}\nonumber \\&-\Delta _\mathrm{v}(1-H_{f})F_{i}, \end{aligned}$$

where $\Delta _{\mathrm{v}}=\delta (\dot{X}_2)$ is the Dirac Delta function, $H_{f}=H(\left| F_i\right| -P_f)$ is the Heaviside function, and $S_{v}=\text {sign}(\dot{X}_2)$ and $S_{f}=\text {sign}(F_i)$ are the sign functions.

Based on the free diagram shown in Fig. 1c, the equations of motion of the capsule system can be written as

8 $$\begin{aligned} \begin{aligned} m_1 \ddot{X}_{1}(t)=&\ P_d\cos (\Omega t)+F_i,\\ m_2 \ddot{X}_{2}(t)=&\ F_i-F_s. \end{aligned} \end{aligned}$$

**Fig. 2:**
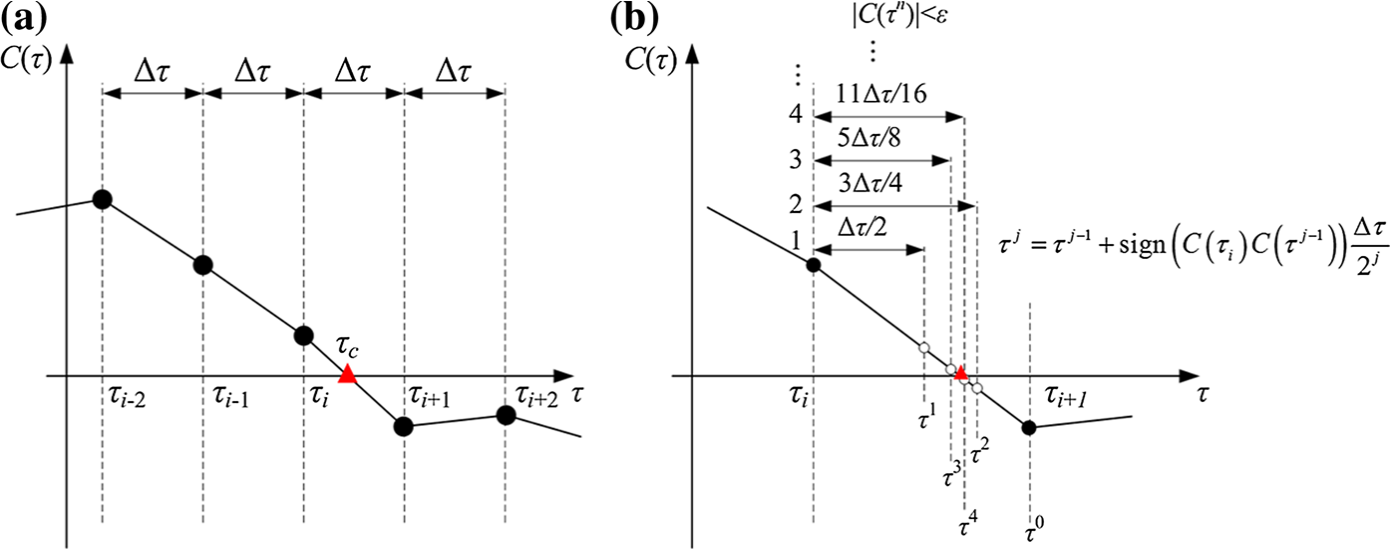
Runge–Kutta method with **a** constant time step and **b** the bisection method for integration. The critical points to be located are marked by *red triangles*. (Color figure online)

**Fig. 3:**
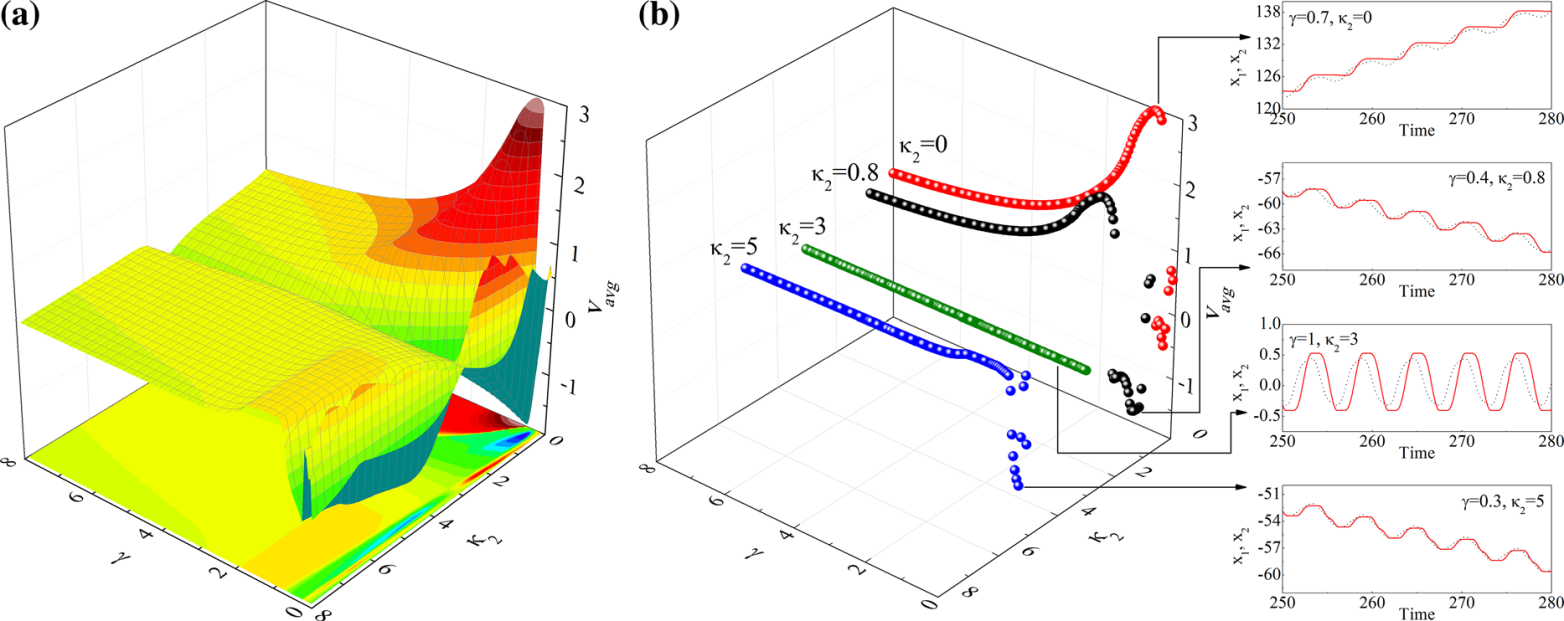
Average progression velocity of the capsule $v_\text {avg}$ under varying mass ratio $\gamma$ and stiffness ratio $\kappa _2$ calculated for $\omega =1.1, \alpha =1.6, \zeta =0.05, \delta _1=0.02, \delta _2=0.02$ and $\kappa _1=3$. The results of calculations are plotted using three-dimensional surface in (**a**) with specific values of spring stiffness $\kappa _2$ presented in (**b**). Additional windows demonstrate the time histories of displacements of the internal mass (*black dash line*) and the capsule (*red solid line*). (Color figure online)

For simplification, we introduce the following non-dimensional parameters

9 $$\begin{aligned} \begin{aligned} \Omega _0&=\sqrt{\frac{k}{m_1}}, \quad \omega =\frac{\Omega }{\Omega _0},\\ \alpha&=\frac{P_d}{P_f}, \quad \zeta =\frac{c}{2m_{1}\Omega _0}, \\ \gamma&=\frac{m_2}{m_1}, \quad \delta _1=\frac{k}{P_f}G_1, \\ \delta _2&=\frac{k}{P_f}G_2,\quad \kappa _1=\frac{k_1}{k},\\ \kappa _2&=\frac{k_2}{k}, \end{aligned} \end{aligned}$$

and variables

10 $$\begin{aligned}&\tau =\Omega _{0}t,\quad x_1=\frac{k}{P_f}X_1,\quad x_2=\frac{k}{P_f}X_2,\nonumber \\&v_1=\frac{\text {d}x_1}{\text {d}\tau }=\frac{k}{\Omega _{0}P_f}\dot{X}_1,\nonumber \\&v_2=\frac{\text {d}x_2}{\text {d}\tau }=\frac{k}{\Omega _{0}P_f}\dot{X}_2. \end{aligned}$$

Then the equations of motion are rewritten as

11 $$\begin{aligned} \begin{aligned} \dot{x}_1(\tau )=&\ v_1(\tau ),\\ \dot{v}_1(\tau )=&\ \alpha \cos (\omega \tau )+f_i,\\ \dot{x}_2(\tau )=&\ v_2(\tau ),\\ \dot{v}_2(\tau )=&\ \frac{1}{\gamma }(f_i+(1-\delta _v)s_{v}+\delta _{v}h_{\!f}s_{\!f}\\&+\delta _{v}(1-h_{\!f})f_i), \end{aligned} \end{aligned}$$

where

$$\begin{aligned} f_i= & {} \ -2\zeta (v_1(\tau )-v_2(\tau ))-(x_1(\tau )-x_2(\tau )) \\&-h_{1}\kappa _{1}(x_1(\tau )-x_2(\tau )-\delta _1)-h_{2}\kappa _{2}(x_1(\tau )\\&-x_2(\tau )+\delta _2), \\ h_1= & {} \ H(x_1(\tau )-x_2(\tau )-\delta _1), \\ h_2= & {} \ H(x_2(\tau )-x_1(\tau )-\delta _2), \\ h_{\!f}= & {} \ H(\left| f_i\right| -1), \\ \delta _v= & {} \ \delta (v_2(\tau )), \\ s_v= & {} \ \text {sign}(v_2(\tau )), \\ s_{\!f}= & {} \ \text {sign}(f_i). \end{aligned}$$

**Fig. 4:**
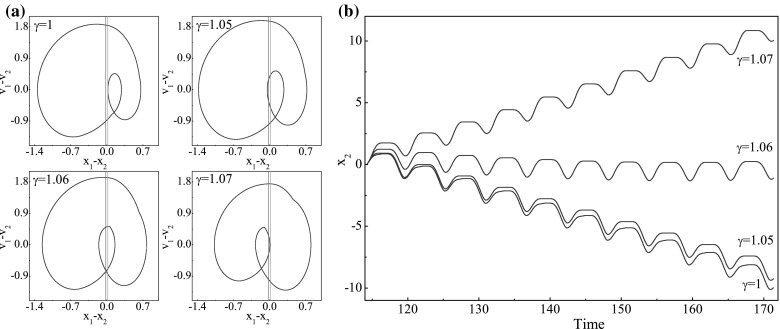
**a** Trajectories on the phase plane ($x_1-x_2, v_1-v_2$) and **b** time histories of displacements of the capsule calculated for $\omega =1.1, \alpha =1.6, \zeta =0.05, \delta _1=0.02, \delta _2=0.02, \kappa _1=3$, and $\kappa _2=0.8$. The locations of the *left* and *right* impact surfaces are shown by *green* and *red lines*, respectively. (Color figure online)

It is worth noting that the non-smooth functions, such as the Heaviside, the Dirac Delta, and the sign functions, have significant influence on simulations. In order to obtain accurate results for numerical simulations, it is important to locate the critical non-smooth points precisely. Here, we adopted the bisection method [30] in the Runge–Kutta simulation, which automatically varies time step to locate the non-smooth points. A schematic illustration of the method is displayed in Fig. 2, where $C(\tau )$ represents the variable of the sign function and $C(\tau _c)=0$ is the switching points (e.g. $C(\tau )=X_1-G_1-X_2=0$ represents the inner mass just impacts the right plate). Figure 2a shows the traditional Runge-Kutta method with constant time step $\Delta \tau$, which skips the critical time instant $\tau _c$ between $\tau _{i}$ and $\tau _{i+1}$. In order to accurately locate $\tau _c$, the bisection algorithm was implemented to adjust the time step once the program detected $C(\tau _{i})C(\tau _{i+1})<0$. With $\tau ^{0}=\tau _{i+1}, \tau ^{j}$ is updated using

12 $$\begin{aligned} \tau ^{j}=\tau ^{j-1}+\text {sign}(C(\tau _{i})C(\tau ^{j-1}))\frac{\Delta \tau }{2^j} \end{aligned}$$

until $C(\tau ^{j})$ is sufficiently close to zero, i.e. $|C(\tau ^{n})|<\epsilon$, where $\epsilon$ is a small positive number given before the simulation. Finally, one can obtain $\tau _c=\tau ^{n}$, which is the critical non-smooth point.

**Fig. 5:**
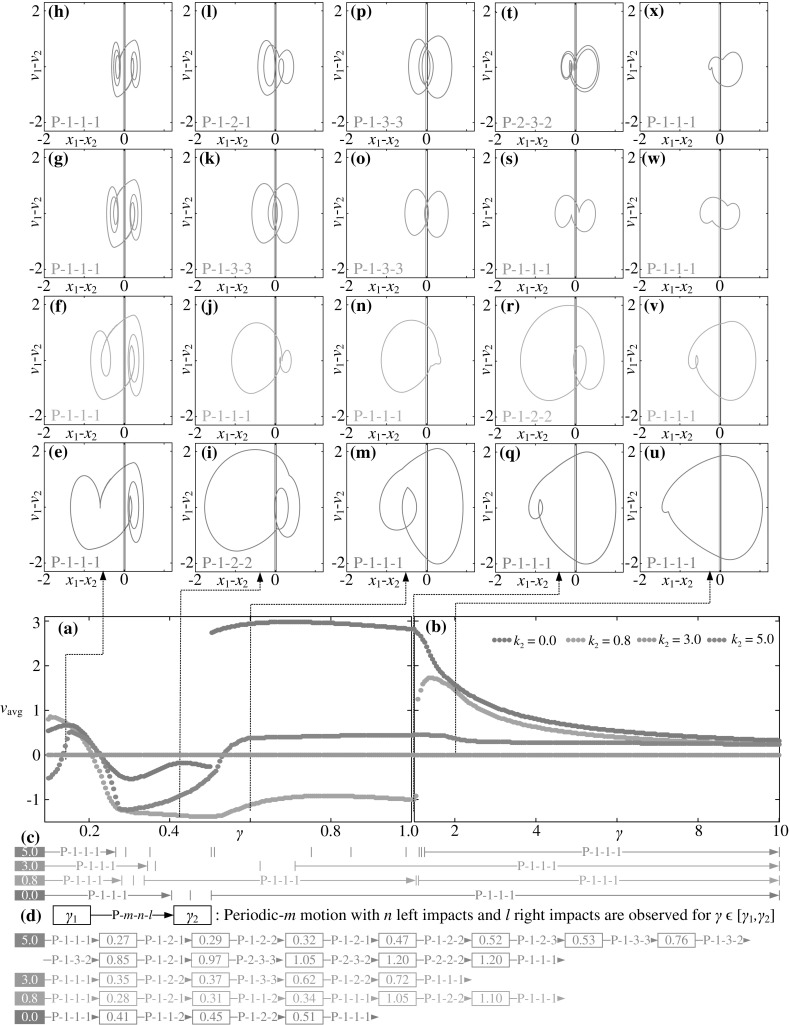
Average progression velocity of the capsule as a function of mass ratio **a** $\gamma \in [0.1,\, 1.0]$ and **b** $\gamma \in [1.0,\, 10]$ calculated for $\omega =1.1, \alpha =1.6, \zeta =0.05, \delta _1=0.02, \delta _2=0.02, \kappa _1=3$ with $\kappa _2=0.0$ (*blue dots*), 0.8 (*orange dots*), 3.0 (*green dots*) and 5.0 (*red dots*). Critical bifurcation points for the capsule system are marked and recorded in (**c**) and (**d**). Additional windows **e**–**u** demonstrate the trajectories of the capsule system on the phase plane. Locations of the left and the right impact surfaces are shown by *purple* and *black lines*, respectively. (Color figure online)

## Bifurcation analysis

In order to gain an understanding of the system dynamics and optimise the progression speed of the capsule, bifurcation analysis is carried out next using the bifurcation diagram where the relative velocity $v^*_1-v^*_2$, which is a projection of the Poincaré map on the $v_1-v_2$ axis, is plotted as a function of the control parameters, including mass ratio, stiffness ratios, gaps of contact, and frequency and amplitude of excitation. The calculations were run for 300 cycles of external excitation, and the data for the first 280 cycles were omitted to ensure steady state response, where the next 20 values of the velocity $v^*_1-v^*_2$ were plotted. The average progression of the capsule

$$\begin{aligned} v_{\text {avg}}=\frac{1}{NT}[x_2(NT)-x_2(0)], \end{aligned}$$

where *N* is the number of cycles and $T=\tfrac{2\pi }{\omega }$ is one period of external excitation, was monitored for the purpose of optimisation. Here, the sign of $v_\text {avg}$ indicates whether the capsule moves forward (positive $v_{\text {avg}}$) or backward (negative $v_{\text {avg}}$). In addition, abbreviations are used to describe periodic motion of the system, e.g. P-1-2-3 represents a period-1 motion with two left impacts and three right impacts per period of external excitation.

**Fig. 6:**
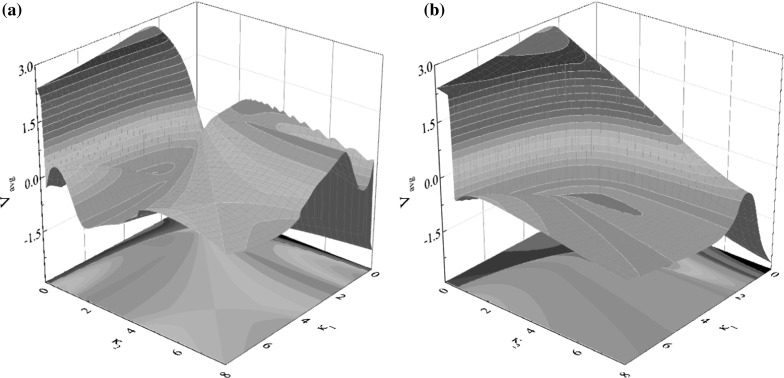
Average progression velocity of the capsule $v_\text {avg}$ under varying the stiffness ratios, $\kappa _1$ and $\kappa _2$ calculated for $\omega =1.1, \alpha =1.6, \zeta =0.05, \delta _1=0.02, \gamma =1$, **a** $\delta _2=0.02$, and **b** $\delta _2=1$

### Influence of mass ratio

To investigate the influence of the mass ratio $\gamma$ and the spring stiffness $\kappa _2$ on the average progression of the capsule, numerical simulations were carried out for $\gamma \in [0,\, 8]$ and $\kappa _2\in [0,\, 8]$, and the calculated results are presented in Fig. 3. It can be seen from Fig. 3a that, the maximum velocity is achieved by the capsule with one-sided constraint ($\kappa _2=0$), and the average velocity of the capsule decreases as the spring stiffness $\kappa _2$ increases. In Fig. 3b, specific values of spring stiffness $\kappa _2$ are shown, where the calculations for $\kappa _2=0,\,0.8,\,3,\,5$ are denoted by red, black, green, and blue, respectively. It can be observed that, except the symmetrical condition ($\kappa _1=\kappa _2=3$), there is an immediate directional change of capsule progression from backward to forward as mass ratio increases, which is due to the grazing when the inner mass contacts with the two-sided constraints. Figure 4 demonstrates the occurrence of such grazing when $\kappa _2=0.8$. It can be seen from Fig. 4a that, the system bifurcates from P-1-1-1 ($\gamma =1$) to P-1-2-2 ($\gamma =1.05$), and then from P-1-2-2 ($\gamma =1.06$) to P-1-2-1 ($\gamma =1.07$). Figure 4b presents the displacements of the capsule from backward to forward progression owing to this grazing-induced bifurcation.

**Fig. 7:**
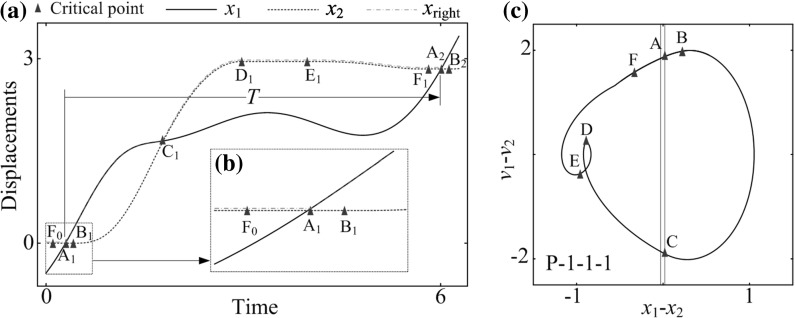
**a** Time histories of the inner mass $x_1$ (*black solid line*), the capsule $x_2$ (*red dash line*), and the right plate $x_\text {right}$ (*blue dash–dot line*), **b** a blow-up window showing the transition from the phase of stationary capsule without contact of the right plate to the phase of stationary capsule with contact, and **c** the trajectory on the phase plane ($x_1-x_2, v_1-v_2$), corresponding to the maximum progression speed obtained for $\omega =1.1, \alpha =1.6, \zeta =0.05, \delta _1=0.02, \delta _2=0.02, \gamma =1, \kappa _1=2.7$, and $\kappa _2=0.0$. Locations of the left and the right impact surfaces are shown by *blue* and *red lines*, respectively. (Color figure online)

**Fig. 8:**
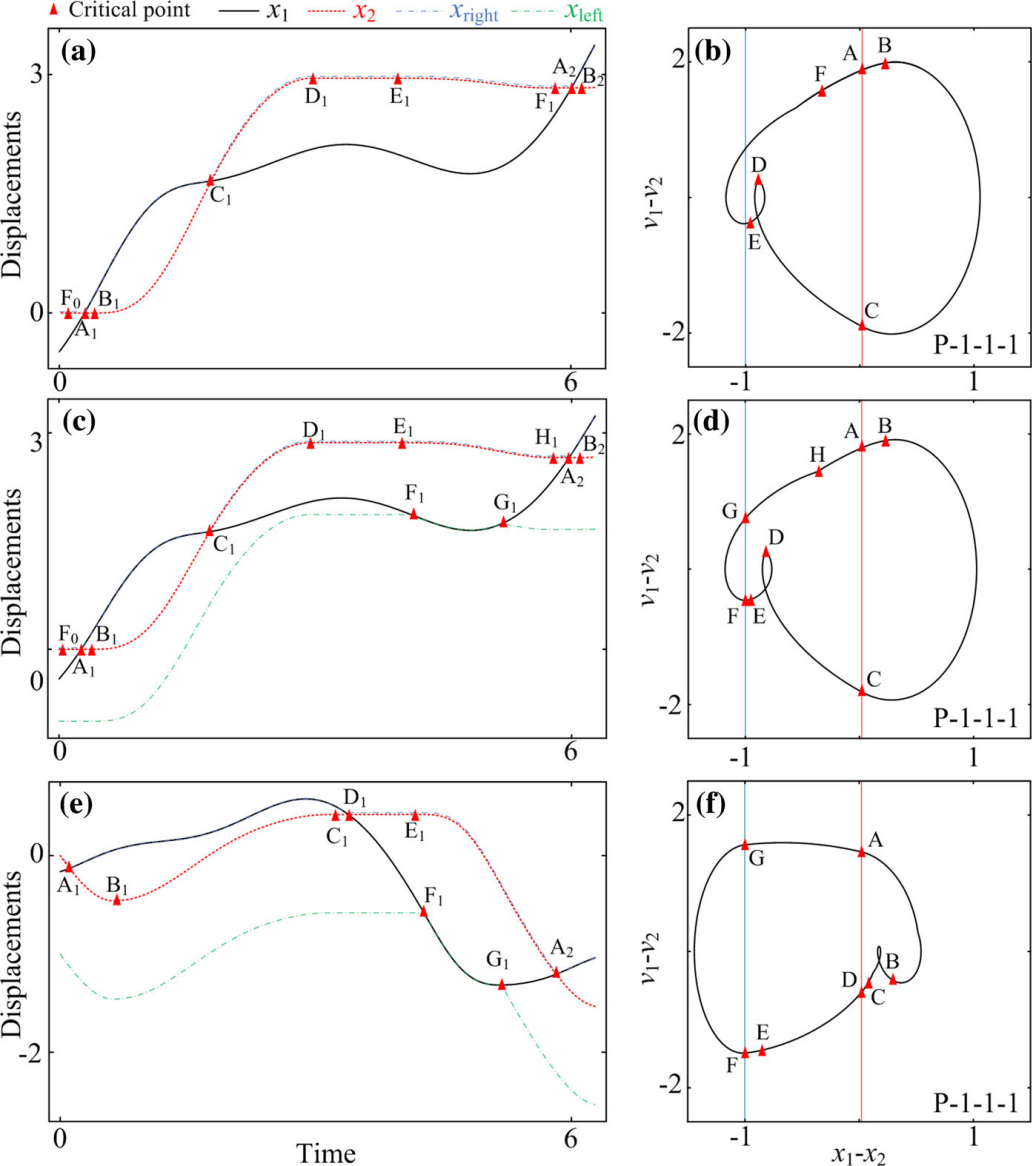
Time histories and phase trajectories of the capsule system computed for $\omega =1.1, \alpha =1.6, \zeta =0.05, \delta _1=0.02, \delta _2=1, \gamma =1, \kappa _1=2.7$, (**a**, **b**) $\kappa _2=0$, (**c**, **d**) $\kappa _2=0.8$, and (**e**, **f**) $\kappa _2=5.0$. Locations of the left and the right impact surfaces are shown by *blue* and *red lines*, respectively. (Color figure online)

**Fig. 9:**
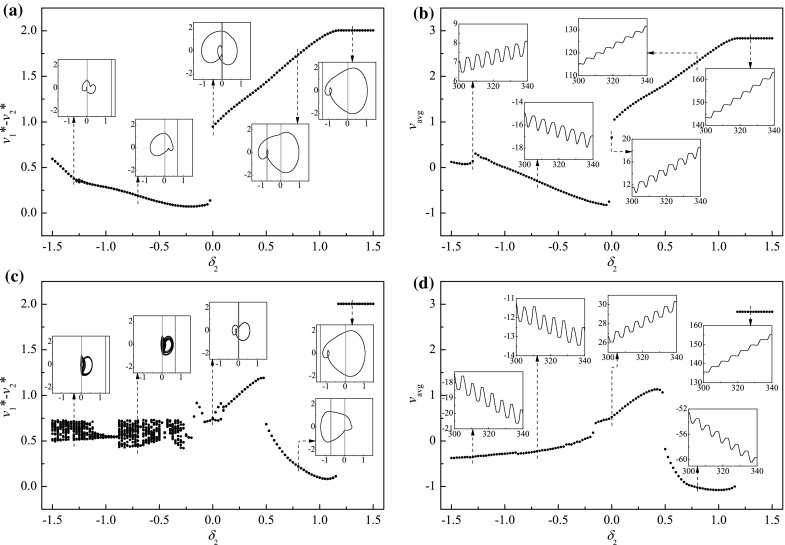
**a** Bifurcation diagram and **b** average progression velocities under variation of gap $\delta _2$ calculated for $\omega =1.1, \alpha =1.6, \zeta =0.05, \kappa _1=2.7, \kappa _2=0.8, \delta _1=0.02, \gamma =1$. **c** Bifurcation diagram and **d** average progression velocities under variation of gap $\delta _2$ calculated for $\omega =1.1, \alpha =1.6, \zeta =0.05, \kappa _1=2.7, \kappa _2=5.0, \delta _1=0.02, \gamma =1$. Additional windows demonstrate the trajectories on the phase plane ($x_1-x_2, v_1-v_2$) and times histories of capsule displacements obtained for $\delta _2=-1.3, \delta _2=-0.7, \delta _2=0, \delta _2=0.8$, and $\delta _2=1.3$. Locations of the impact surfaces for the right and the left constraints are shown by *red* and *blue lines*, respectively. (Color figure online)

**Fig. 10:**
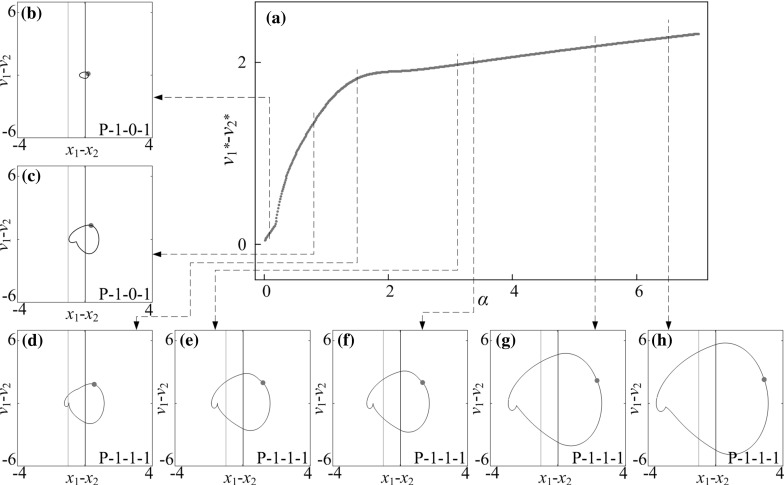
**a** Bifurcation diagram under variation of amplitude of excitation $\alpha$ calculated for $\omega =1.1, \zeta =0.05, \gamma =1, \delta _1=0.02, \delta _2=1.0, \kappa _1=2, \kappa _2=0$. **b**–**h** Trajectories of the system displayed on the phase plane ($x_1-x_2, v_1-v_2$) for $\alpha =0.10, 0.80, 1.50, 3.12, 3.37, 5.43$ and 6.50. Locations of the left and the right impact surfaces are shown by *purple* and *red lines*, respectively. Poincaré sections are marked by blue dots on the phase plane. (Color figure online)

**Fig. 11:**
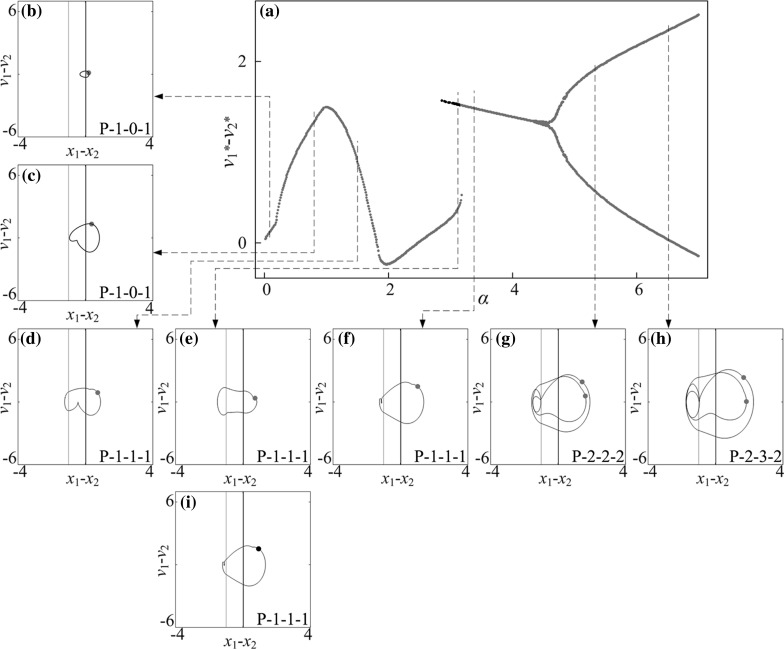
**a** Bifurcation diagram under variation of amplitude of excitation $\alpha$ calculated for $\omega =1.1, \zeta =0.05, \gamma =1, \delta _1=0.02, \delta _2=1.0, \kappa _1=2, \kappa _2=5$. **b**–**i** Trajectories of the system displayed on the phase plane ($x_1-x_2, v_1-v_2$) for $\alpha =0.10, 0.80, 1.50, 3.12, 3.37, 5.43$ and 6.50. Coexisting attractors are marked by *red dots*. Locations of the left and the right impact surfaces are shown by *purple* and *red lines*, respectively. Poincaré sections are marked by orange and red dots on the phase plane. (Color figure online)

A further investigation of influence of the mass ratio $\gamma$ and the spring stiffness $\kappa _2$ on the average velocity of the capsule was carried out, and the calculated results are shown in Fig. 5. As can be seen from these figures, at $\gamma =0.1$, Fig. 5e–h correspond to $\kappa _2=0.0, 0.8, 3.0$ and 5.0, respectively. The corresponding motions are all P-1-1-1 and the capsule moves forward. Increasing the mass ratio to 0.45, one obtains negative velocities with the capsule motions becoming P-1-2-2, P-1-1-1, P-1-3-3, and P-1-2-1 as illustrated in Fig. 5i–k and (l), respectively. Here, although they are all period-1 motions, the number of impacts per period of external excitation increases. For example, for $\kappa _2=3$, the capsule system bifurcates from P-1-1-1 to P-1-3-3 as the mass ratio increases from 0.1 to 0.45. It can be observed from Fig. 5a that, as the mass ratio further increases, the average velocity of the capsule increases drastically and achieves its maximum for the capsule with one-sided constraint ($\kappa _2=0.0$), while the average velocity of the capsule experiences a continuous rise to a small positive value when the left spring is stiffer ($\kappa _2=5.0$). However, such phenomenon cannot be observed when the stiffness of the left spring is weak ($\kappa _2=0.8$) or symmetrical ($\kappa _2=3.0$). When $\kappa _2=0.8$, the drastic change of the average velocity from negative to positive is recorded at $\gamma \approx 1.06$. By comparing Fig. 5i with (m) and (r) with (v), one may notice that both drastic changes are due to grazing bifurcations as illustrated in Fig. 4. From Fig. 5t, we can observe a period-2 motion (P-2-3-2) which indicates that stiffer spring may induce period doubling of the capsule system. As the mass ratio increases, the inner mass has less effect on the dynamics of the capsule, so all the velocities gradually decline to zero regardless of the values of $\kappa _2$. In addition, it is worth noting that, by comparing all the trajectories on the phase plane in Fig. 5, the left spring can effectively constrain the stroke length of the inner mass when its stiffness is sufficiently large.

From a control point of view, it is more effective to control the direction of capsule progression when the weight of the capsule is less than or equal to the weight of the internal mass ($\gamma \le 1$) providing that the two-sided constraints are asymmetrical ($\kappa _1\ne \kappa _2$). The control strategy for the capsule system could be to alter its mass ratio slightly around $\gamma =1$ for controlling its progression direction by using a weak left constraint (e.g. $\kappa _2=0.8$), which offers both forward and backward progressions at acceptable average speeds.

### Influence of stiffness ratios

Average progression velocity of the capsule under varying the stiffness ratios, $\kappa _1$ and $\kappa _2$ are presented in Fig. 6a, where the gaps of contact for both springs are equal ($\delta _1=\delta _2=0.02$). As can be seen from this figure, the best progression is achieved by the capsule system with one-sided constraint ($\kappa _2=0$), and the maximal average velocity is recoded at $\kappa _1=2.7$ and $\kappa _2=0$. Figure 7a shows the time histories of displacements of the inner mass and the capsule, and Fig. 7c presents the capsule trajectory on the phase plane ($x_1-x_2, v_1-v_2$) for the maximal average velocity recorded in Fig. 6a. A blow-up window in Fig. 7b clearly shows the displacements of the mass, the capsule, and the right plate transiting from the phase of stationary capsule without contact of the right plate to the one with contact. It can be seen that the maximum speed is achieved by P-1-1-1, and an entire time period (*T*) of the motion has been divided into six phases. The first phase begins at Point $\text {A}$, where the inner mass just contacts with the right plate, but the elastic forces on the capsule are too small to overcome external frictional resistance until Point $\text {B}$ is reached. Thereafter, the capsule accelerates to progress before Point $\text {C}$, where the mass is separated from the right plate and the capsule is decelerated by the friction. The velocity of the capsule reduces to zero at Point $\text {D}$ and the capsule is stationary until Point $\text {E}$. At Point $\text {E}$, the elastic forces on the capsule exceed the threshold of the static friction, and a slight backward motion of the capsule is observed. At Point $\text {F}$, the velocity of the capsule becomes zero again, and the capsule has a short period of sticking phase until Point $\text {A}$.

The capsule progression for unsymmetrical gaps of contact ($\delta _1=0.02$ and $\delta _2=1$) was studied, and the calculated result is presented in Fig. 6b. It can be seen from the figure that one-sided constraint (i.e. $\kappa _2=0$ or $\kappa _1=0$) is the best choice for both forward and backward progressions. A detailed behaviour of the capsule system are shown in Fig. 8, where the capsules with one-sided constraint ($\kappa _2=0$), two-sided constraints with weak left spring ($\kappa _2=0.8$), and two-sided constraints with strong left spring ($\kappa _2=5$) are plotted in Fig. 8. Comparing the cases for one-sided and two-sided constraints with weak left spring, the trajectories in Fig. 8a–d are similar until Point $\text {F}$ at where the inner mass contacts with the left plate and the capsule has a slightly larger backward displacement thereafter. Therefore, the velocity of the capsule can be lowered by inducing the left spring. Moreover, when the stiffness of the left spring becomes greater ($\kappa _2=5.0$), large backward progression of the capsule encounters, and the overall progression of the capsule becomes negative as shown in Fig. 8e, f.

**Fig. 12:**
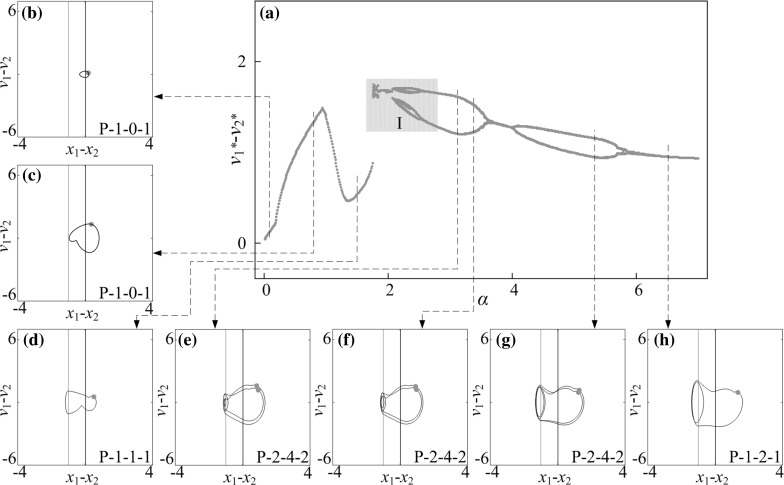
**a** Bifurcation diagram under variation of amplitude of excitation $\alpha$ calculated for $\omega =1.1, \zeta =0.05, \gamma =1, \delta _1=0.02, \delta _2=1.0, \kappa _1=2, \kappa _2=20$. **b**–**h** Trajectories of the system displayed on the phase plane ($x_1-x_2, v_1-v_2$) for $\alpha =0.10, 0.80, 1.50, 3.12, 3.37, 5.43$ and 6.50. Locations of the left and the right impact surfaces are shown by *purple* and *red lines*, respectively. Poincaré sections are marked by green dots. (Color figure online)

An investigation on the gap of left constraint was carried out, and the comparison between the weak left constraint ($\kappa _2=0.8$) and the strong one ($\kappa _2=5.0$) is shown in Fig. 9. Here, we calculated the gap of the left constraint for $\delta _2\in [-1.5,\,1.5]$, where the negative value of the gap indicates a prestressed constraint. As can be seen from the figure, when the gap decreases from $\delta _2=1.5$, the internal mass begins to impact left constraint at $\delta _2=1.175$. As shown in Fig. 9b, d, the grazing of such impact alters the direction of capsule progression from forward to backward for the capsule with strong left constraint, while it only affects the forward speed of the capsule with weak left constraint. As the gap decreases, the capsule with weak left constraint encounters this sudden change of progression direction owing to the grazing contact with the left constraint at $\delta _2\approx 0$. On the other hand, the capsule with strong left constraint experiences a period and a reverse period doubling at $\delta _2=0.125$ and $-0.125$, respectively. When the left constraint is prestressed ($\delta _2<0$), comparing Fig. 9a, c, the capsule with weak constraint has period-1 motion, while the one with strong constraint experiences chaotic motion. It can be found from Fig. 9b ,d, both average progressions are slow. However, as presented in the additional windows of phase trajectories, the stroke length of the internal mass under prestressed condition is significantly reduced comparing to the one with right constraint only.

Based on the discussions above, it can be drawn that the maximum positive progression velocity can be achieved by the capsule with one-sided right constraint, and the introduction of the left spring with any value of stiffness may reduce its progression speed. Nevertheless, if the miniaturization of the capsule is required, the prestressed weak constraint could be a viable option.

**Fig. 13:**
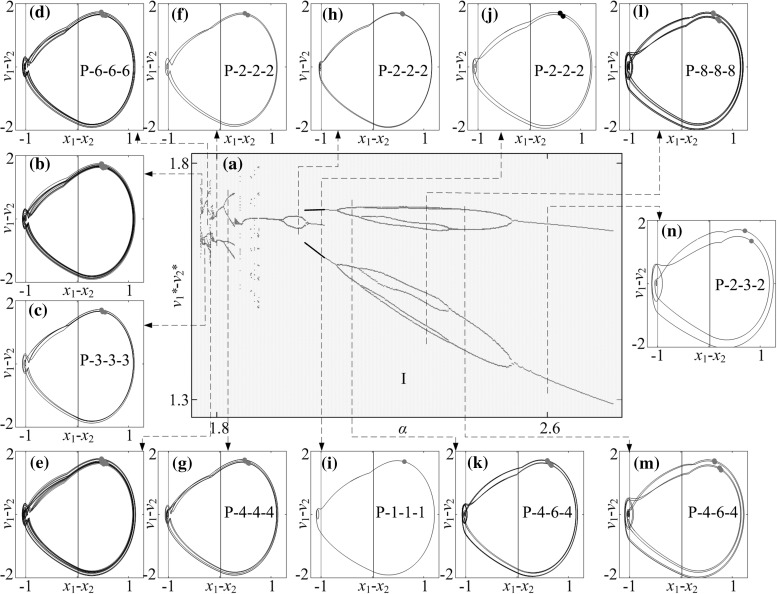
**a** Bifurcation diagram in the range marked by the number “I” in Fig. 12, where coexisting attractors are marked by *red dots*. **b**–**n** Additional windows demonstrate the trajectories on the phase plane ($x_1-x_2, v_1-v_2$) obtained for $\alpha =1.763, 1.768, 1.780, 1.785, 1.8, 1.820, 2.0, 2.056, 2.056$ (coexisting), 2.123, 2.312, 2.410, and 2.6. Locations of the left and the right impact surfaces are shown by *blue* and *red lines*, respectively. Poincaré sections are marked by green and red dots on the phase plane. (Color figure online)

**Fig. 14:**
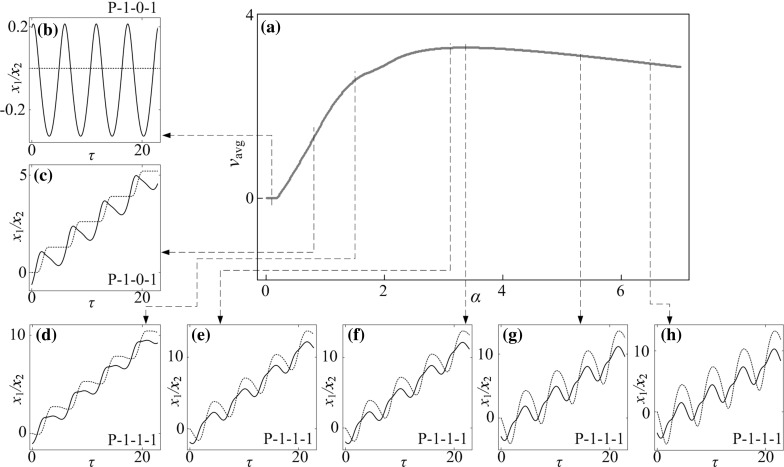
**a** Average progression velocities under variation of amplitude of excitation $\alpha$ calculated for $\omega =1.1, \zeta =0.05, \gamma =1, \delta _1=0.02, \delta _2=1.0, \kappa _1=2, \kappa _2=0$. **b**–**h** Additional windows demonstrate the time histories of displacements of the inner mass (*black solid line*) and the capsule (*red dash line*) obtained for $\alpha =0.10, 0.80, 1.50, 3.12, 3.37, 5.43$ and 6.50. (Color figure online)

**Fig. 15:**
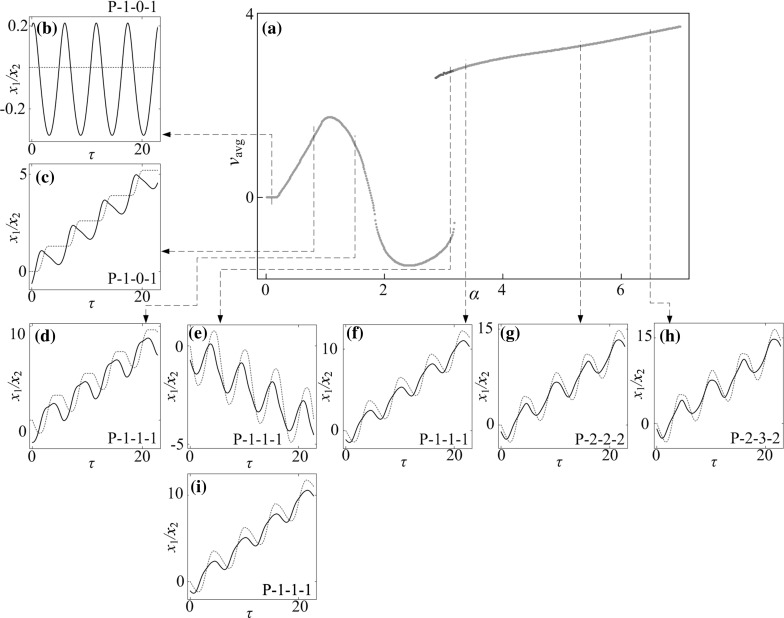
**a** Average progression velocities under variation of amplitude of excitation $\alpha$ calculated for $\omega =1.1, \zeta =0.05, \gamma =1, \delta _1=0.02, \delta _2=1.0, \kappa _1=2, \kappa _2=5$. **b**–**i** Additional windows demonstrate the time histories of displacements of the inner mass (*black solid line*) and the capsule (*red dash line*) obtained for $\alpha =0.10, 0.80, 1.50, 3.12, 3.37, 5.43$ and 6.50. Coexisting attractors are indicated by *red dots*. (Color figure online)

### Influence of amplitude of excitation

Bifurcation diagrams of the capsule system with $\kappa _2=0,\, 5,\, 20$ under variation of amplitude of excitation $\alpha$ are presented in Figs. 10, 11, and 12, respectively. Additional windows in these figures demonstrate the capsule trajectories on the phase plane ($x_1-x_2, v_1-v_2$). One can observe that the bifurcation diagram shown in Fig. 10 for the capsule with one-sided constraint ($\kappa _2=0$) is smooth, and all its corresponding phase trajectories are P-1-1-1. For $\kappa _2=5$ shown in Fig. 11, the bifurcation curve is smooth until $\alpha =2.86$ at where an immediate increase in the relative velocity $v_{1}-v_{2}$ due to the grazing contact with the left spring is recorded, and two P-1-1-1 presented in Fig. 11e, i coexist for $\alpha \in [2.86, 3.18]$. As the amplitude of excitation increases, a period doubling is observed at $\alpha =4.35$ and the capsule bifurcates from P-1-1-1 to P-2-2-2. When the left spring is much stiffer ($\kappa _2=20$), the dynamic responses of the system become more complicated. The grazing event of the left plate is recorded at $\alpha =1.76$ followed by a period doubling cascade, which is shown in Fig. 12. As the amplitude of excitation increases, a reverse period doubling from P-2-4-2 to P-1-2-1 is observed at $\alpha =3.69$. Thereafter, a period and a reverse period doubling between P-2-4-2 and P-1-2-1 are recorded at $\alpha =4$ and 5.87, respectively.

**Fig. 16:**
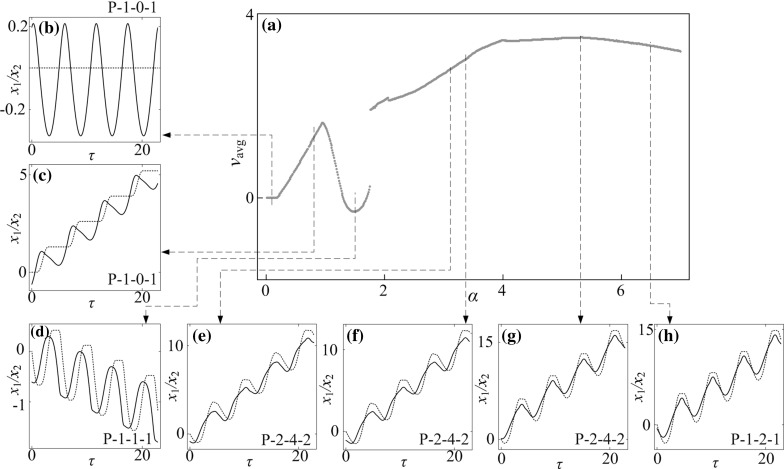
**a** Average progression velocities under variation of amplitude of excitation $\alpha$ calculated for $\omega =1.1, \zeta =0.05, \gamma =1, \delta _1=0.02, \delta _2=1.0, \kappa _1=2, \kappa _2=20$. **b**–**h** Additional windows demonstrate the time histories of displacements of the inner mass (*black solid line*) and the capsule (*red dash line*) obtained for $\alpha =0.10, 0.80, 1.50, 3.12, 3.37, 5.34$ and 6.50. (Color figure online)

In order to obtain a better insight into the dynamic responses of the stiffer left spring ($\kappa _2=20$), Fig. 13 presents the bifurcations in the range marked by the number “I” in Fig. 12. As can be seen from this figure, the system starts with a quasi-periodic motion which is followed by a period-3 and period-6 motions. As the amplitude of excitation increases, another quasi-periodic motion followed by a period-2 and period-4 motions is observed. For a short range of amplitude of excitation, the coexistence of a P-1-1-1 and a P-2-2-2 motion is recorded where coexisting attractors are marked by red dots. From this point onwards, the capsule bifurcates from P-2-2-2 to P-4-6-4 and to P-8-8-8 both via a period doubling at $\alpha =2.323$ and 2.362, respectively. Thereafter, the capsule experiences two reverse period doublings at $\alpha =2.323$ and 2.362, consequently, the dynamic response of the system bifurcates from P-8-8-8 to P-2-3-2, and then P-2-4-2 afterwards.

The calculated average velocities of the capsule with $\kappa _2=0,\, 5,\, 20$ under variation of amplitude of excitation $\alpha$ are presented in Figs. 14, 15 and 16, respectively, with additional windows demonstrating the time histories of displacements of the inner mass (black solid line) and the capsule (red dash line). It can be seen from these figures that, the average velocities vary with respect to the increase of excitation amplitude. When the excitation amplitude is small, the capsule is stationary owing to the static friction from the supporting surface. As the excitation amplitude increases, all the systems experience P-1-0-1 responses with the same progression speeds until the inner mass firstly contacts with the left constraint at $\alpha =0.93$. Due to the effect of the left constraint, the capsule systems with two-sided constraints ($\kappa _2=5$ and 20) have lower average speeds than the one with one-sided constraint. Comparing their displacements in Figs. 14d, 15d and 16d, it can be noted that increase of stiffness of the left spring may cause fluctuation of the inner mass leading to backward motion of the capsule. It is worth noting that a forward P-1-1-1 coexists with a backward P-1-1-1 for a short range of amplitude of excitation for $\kappa _2=5$ due to the grazing contact with the left constraint. For one-sided constraint ($\kappa _2=0$), the maximum velocity of the capsule is achieved by a P-1-1-1 motion at $\alpha =3.37$. For $\kappa _2=20$, the capsule with two-sided constraints reaches its maximum velocity at $\alpha =5.34$ by a P-2-4-2 motion, which is higher than the system with one-sided constraint. With a weak left spring ($\kappa _2=5$), it can be seen from Fig. 15h that, the maximal average speed obtained by the capsule through a P-2-3-2 motion is higher than the maximal speeds recorded for the other two systems. Here, it can be concluded that the best performance of the capsule system could be achieved by using a weak left spring under a larger amplitude of excitation.

**Fig. 17:**
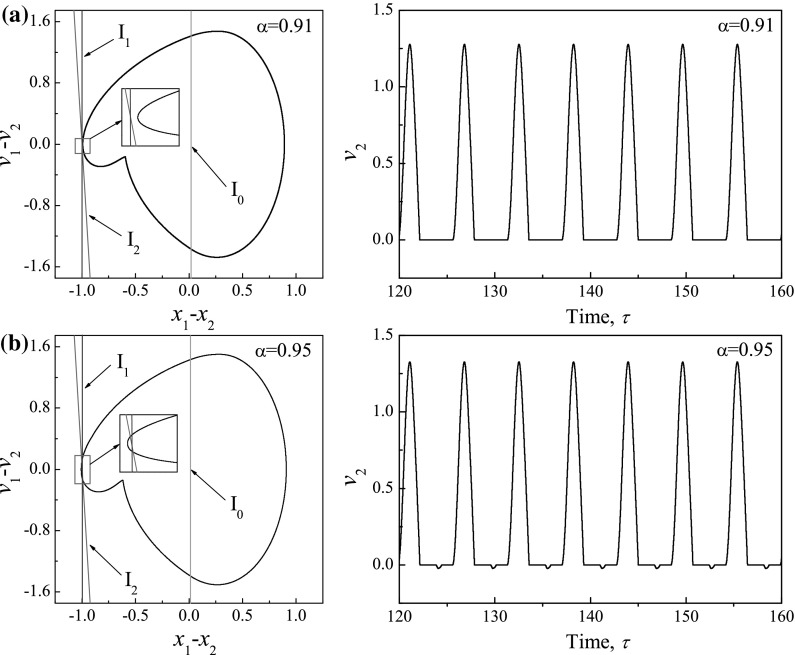
Trajectories on the phase plane ($x_1-x_2, v_1-v_2$) and time histories of velocities of the capsule, $v_2$ obtained for $\omega =1.1, \zeta =0.05, \gamma =1, \delta _1=0.02, \delta _2=1.0, \kappa _1=2, \kappa _2=20$: **a** $\alpha =0.91$ (*before grazing*) and **b** $\alpha =0.95$ (*after grazing*), where $I_0:=(x_1-x_2)-\delta _1=0$ (*red lines*), $I_1:=(x_1-x_2)+\delta _2=0$ (*blue lines*), and $I_2:=(x_1-x_2)+2\zeta (v_1-v_2)+\kappa _2(x_1-x_2+\delta _2)+1=0$ (*green lines*). (Color figure online)

**Fig. 18:**
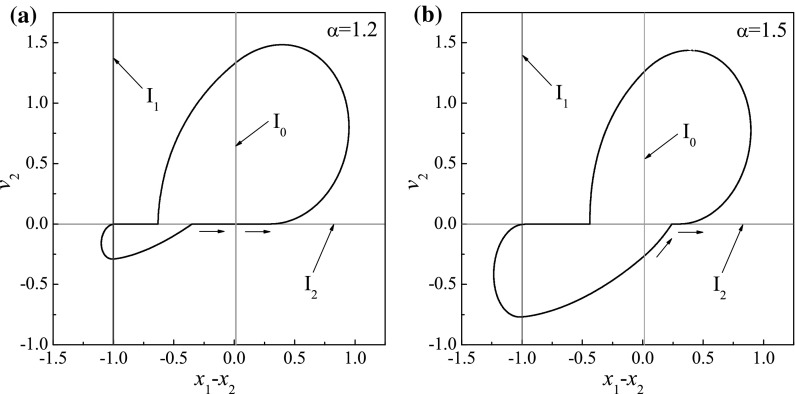
Trajectories on the phase plane ($x_1-x_2, v_2$) computed for $\omega =1.1, \zeta =0.05, \gamma =1, \delta _1=0.02, \delta _2=1.0, \kappa _1=2, \kappa _2=5$: **a** $\alpha =1.2$ (*before* the boundary-intersection crossing bifurcation) and **b** $\alpha =1.5$ (*after* the boundary-intersection crossing bifurcation), where $I_0:=(x_1-x_2)-\delta _1=0$ (*red lines*), $I_1:=(x_1-x_2)+\delta _2=0$ (*blue lines*), and $I_2:=v_2=0$ (*green lines*). (Color figure online)

Figure 17 shows an interesting grazing bifurcation observed for $\kappa _2=20$ when the phase trajectory of the system makes grazing contact simultaneously with two discontinuity boundaries, $I_1:=(x_1-x_2)+\delta _2=0$ and $I_2:=(x_1-x_2)+2\zeta (v_1-v_2)+\kappa _2(x_1-x_2+\delta _2)+1=0$, which define the impact of the left constraint and the transition to backward drift, respectively. As can be seen from Fig. 17a, the capsule has forward drift only when its phase trajectory does not contact the discontinuity boundaries at $\alpha =0.91$. As the amplitude of excitation increases to $\alpha =0.95$, two grazing contacts occur and the backward drift of the capsule appears at every cycle. Another interesting bifurcation, namely the boundary-intersection crossing bifurcation [19], is observed for $\kappa _2=5$ which is presented in Fig. 18. One can observe from Fig. 18a that, when the capsule is stationary $I_2:=v_2=0$, the trajectory of the capsule on the phase plane ($x_1-x_2, v_2$) crosses the discontinuity boundary, $I_0:=(x_1-x_2)-\delta _1=0$, which defines the impact of the right constraint. As shown in Fig. 18b, when the amplitude of excitation increases to $\alpha =1.5$, the trajectory hits the discontinuity boundary $I_0$ at when the capsule has backward drift (i.e. $v_2<0$). Identification of both bifurcations are very important for the capsule system, since avoidance of such events may reduce the energy loss caused by external friction, so that improving energy efficiency of the system.

**Fig. 19:**
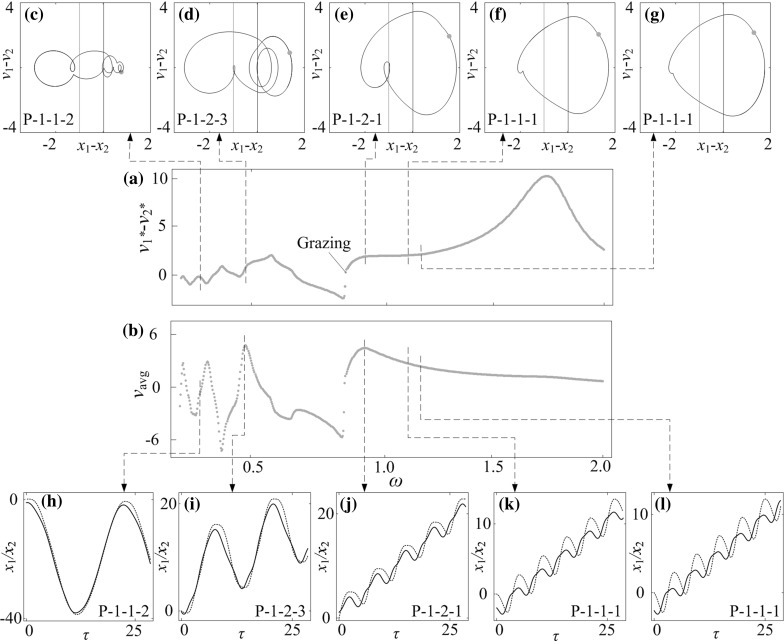
**a** Bifurcation diagram and **b** average progression velocities of the capsule system under variation of frequency of excitation $\omega$ calculated for $\alpha =3.37, \zeta =0.05, \gamma =1, \delta _1=0.02, \delta _2=1.00, \kappa _1=2, \kappa _2=0$. **c**–**g** Trajectories of the system displayed on the phase plane ($x_1-x_2, v_1-v_2$), and **h**–**l** time histories of displacements of the inner mass (*black solid lines*) and the capsule (*red dash line*) obtained for $\omega =0.284, 0.479, 0.986, 1.169$, and 1.226. Locations of the left and the right impact surfaces are shown by *purple* and *red lines*, respectively. Poincaré sections are marked by blue dots on the phase plane. (Color figure online)

**Fig. 20:**
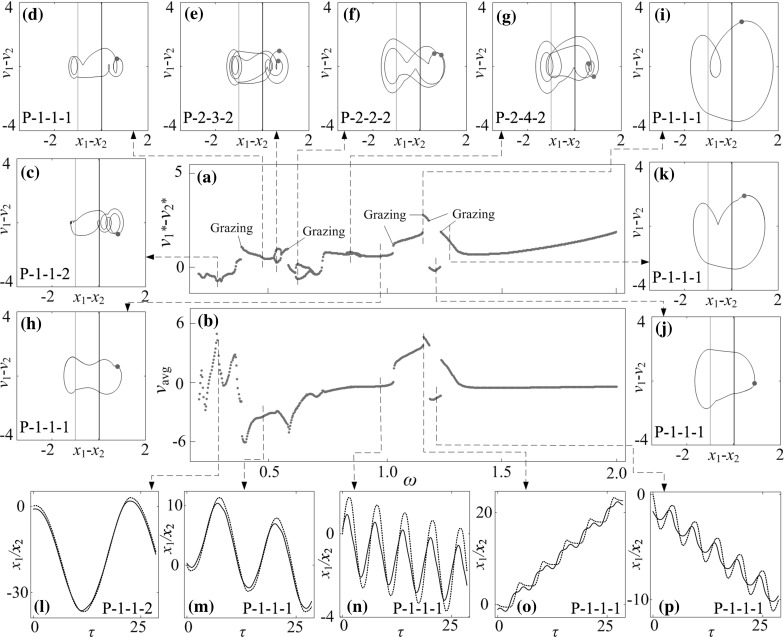
**a** Bifurcation diagram and **b** average progression velocities of the capsule system under variation of frequency of excitation $\omega$ calculated for $\alpha =3.37, \zeta =0.05, \gamma =1, \delta _1=0.02, \delta _2=1.00, \kappa _1=2, \kappa _2=5$. **c**–**k** Trajectories of the system displayed on the phase plane ($x_1-x_2, v_1-v_2$) obtained for $\omega =0.284, 0.479, 0.548, 0.629, 0.854, 0.986, 1.169, 1.226$, and 1.244. Locations of the left and the right impact surfaces are shown by *purple* and *red lines*, respectively. **l**–**p** Time histories of displacements of the inner mass (*black solid lines*) and the capsule (*red dash line*) obtained for $\omega =0.284, 0.479, 0.986, 1.169$, and 1.226. Poincaré sections are marked by orange dots on the phase plane. (Color figure online)

**Fig. 21:**
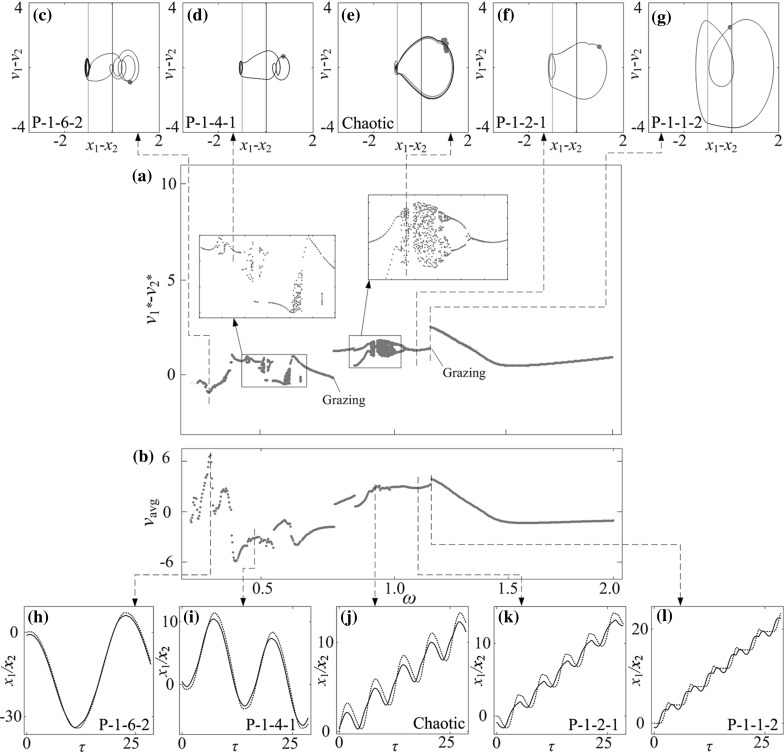
**a** Bifurcation diagram and **b** average progression velocities of the capsule under variation of frequency of excitation $\omega$ calculated for $\alpha =3.37, \zeta =0.05, \gamma =1, \delta _1=0.02, \delta _2=1.00, \kappa _1=2, \kappa _2=20$. **c**–**g** Trajectories of the system displayed on the phase plane ($x_1-x_2, v_1-v_2$), and **h**–**l** time histories of displacements of the inner mass (*black solid line*) and the capsule (*red dash line*) obtained for $\omega =0.284, 0.479, 0.986, 1.169$, and 1.226. Locations of the left and the right impact surfaces are shown by *purple* and *red lines*, respectively. Poincaré sections are marked by green dots on the phase plane. (Color figure online)

### Influence of frequency of excitation

The effect of frequency of excitation $\omega$ on capsule dynamics is studied in this section. Bifurcation diagrams and average progressions of the capsule computed for $\kappa _2=0, 5$, and 20 are presented, respectively, in Figs. 19, 20 and 21 with additional panels illustrating phase trajectories and time histories of displacements of the inner mass and the capsule. In general, when the frequency of excitation is low ($\omega <0.5$), the phase trajectories of the capsule system are twisted and several impacts are encountered as illustrated in Figs. 19c, d, 20c, d and 21c, d. When the frequency is sufficiently large ($\omega >1.5$), the dynamic responses of the capsule are mainly period-1 motions. Comparing the bifurcation diagrams in Figs. 19a, 20a, and 21a, one can see that, the response of the capsule for $\kappa _2=0$ is mainly period-1 motion, and an immediate change of capsule direction due to grazing contact is recorded. As $\kappa _2$ increases, the dynamics of the capsule system becomes complex. For example, when $\kappa _2=5$, a number of grazing bifurcations, periodic doublings, and reverse period doublings are recorded. These grazing singularities induce sudden jumps of average progression velocities of the capsule at $\omega =0.384, 0.585, 1.036, 1.170, 1.197$, and 1.244, and the jump at $\omega =0.1169$ yields the fastest progression velocity for $\kappa _2=5$, which is shown in Fig. 20i. In addition, the period doubling leads to period-2 motion for $\omega \in [0.525, 0.561], [0.616, 0.687]$ and [0.834, 0.900]. Phase trajectories of three selected periodic-2 responses within these parameter regions are plotted in Fig. 20e–g. When $\kappa _2=20$, the bifurcation pattern shown in Fig. 21a becomes more complex. Two blow-up windows were plotted to show more details of these bifurcations. The first window shows the switches between period-1 and chaotic motions through several grazing contacts, while the second one illustrates the successive reverse period doublings from chaotic to period-1 response.

Comparing the average velocities of the capsules with different $\kappa _2$, it can be found that, when the frequency of excitation is low ($\omega <0.5$), any small variations of excitation frequency may affect the average speed significantly. As can be seen from Fig. 19b, two local peaks are recorded at $\omega =0.479$ and 0.986 for a P-1-2-3 and a P-1-2-1 motion, respectively. The maximal average velocity for $\kappa _2=5$ is achieved by a P-1-1-1 motion at $\omega =1.169$. For $\kappa _2=20$, the maximal velocity is achieved by a P-1-1-2 motion at $\omega =1.226$. Comparing these two motions presented in Figs. 20o and 21l, both inner masses have two phases of forward motion in each period of excitation, and the backward motions of both capsules are stopped by the second forward phase of the inner mass. As a result, the progression velocities for both capsules are improved.

## Energy consumption and cabin length

**Fig. 22:**
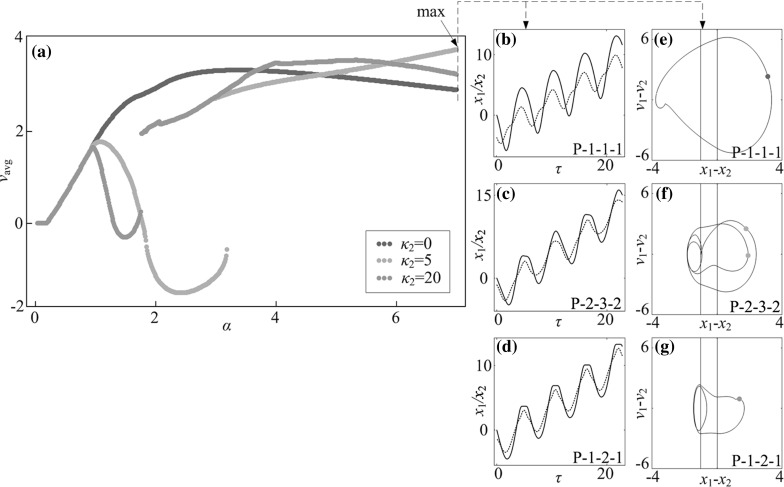
**a** Average progression velocities of the capsule systems with $\kappa _2=0$ (*blue dots*), $\kappa _2=5$ (*orange dots*), and $\kappa _2=20$ (*green dots*) are plotted as functions of amplitude of excitation $\alpha$ calculated for $\omega =1.1, \zeta =0.05, \gamma =1, \delta _1=0.02, \delta _2=1.0$, and $\kappa _1=2$. Additional windows demonstrate the time histories of displacements of the inner mass (*black solid line*) and the capsule (*red dash line*) obtained for **b** $\kappa _2=0$, **c** 5, **d** 20, and the trajectories on the phase plane ($x_1-x_2, v_1-v_2$) obtained for **e** $\kappa _2=0$, **f** 5, **g** 20. Locations of the left and the right impact surfaces are shown by *blue* and *red lines*, respectively. Poincaré sections are marked by blue, orange, and green dots on the phase plane. (Color figure online)

In this section, we will study the best control parameters for capsule progression. In Fig. 15, the fastest speed of the capsule is achieved by the system with two-sided constraints ($\kappa _2=5$). Therefore, as shown in Fig. 22c, f, an optimum approach to control capsule progression is to increase the amplitude of excitation as large as possible. However, larger amplitude consumes more energy, so that the control parameters for the fastest progression are not the most efficient ones [8]. In order to consider this, we introduce the normalised average velocity of the capsule $v_\text {E}$, which is given as

13 $$\begin{aligned} v_\text {E}=\frac{N v_\text {avg}}{\int _0^{NT}\alpha \cos (\omega \tau ) v_1(\tau )\text {d}\tau }. \end{aligned}$$

**Fig. 23:**
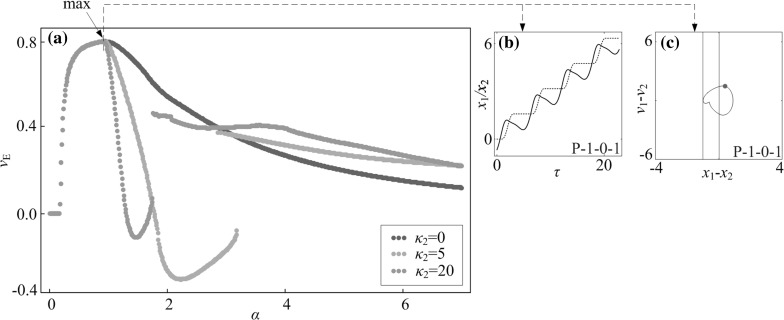
**a** Average progression velocities per energy consumption with $\kappa _2=0$ (*blue dots*), $\kappa _2=5$ (*orange dots*), and $\kappa _2=20$ (*green dots*) are plotted as functions of amplitude of excitation $\alpha$ calculated for $\omega =1.1, \zeta =0.05, \gamma =1, \delta _1=0.02, \delta _2=1.0$, and $\kappa _1=2$. Additional windows demonstrate **b** the time histories of displacements of the inner mass (*black solid line*) and the capsule (*red dash line*) and **c** the trajectories on the phase plane ($x_1-x_2, v_1-v_2$) obtained for $\alpha =0.92$, where the maximal $v_\text {E}$ is achieved. Locations of the left and the right impact surfaces are shown by *blue* and *red lines*, respectively. Poincaré sections are marked by blue dots on the phase plane. (Color figure online)

Based on Eq. (13), the results in Fig. 22 were recalculated and the new results are shown in Fig. 23. It is seen that the maximal $v_\text {E}$ is achieved at $\alpha =0.92$ through a P-1-0-1 motion. In other words, the capsule system with one-sided constraint has the most energy-efficient progression. As the amplitude of excitation $\alpha$ increases, the normalised average velocity $v_\text {E}$ drops dramatically.

**Fig. 24:**
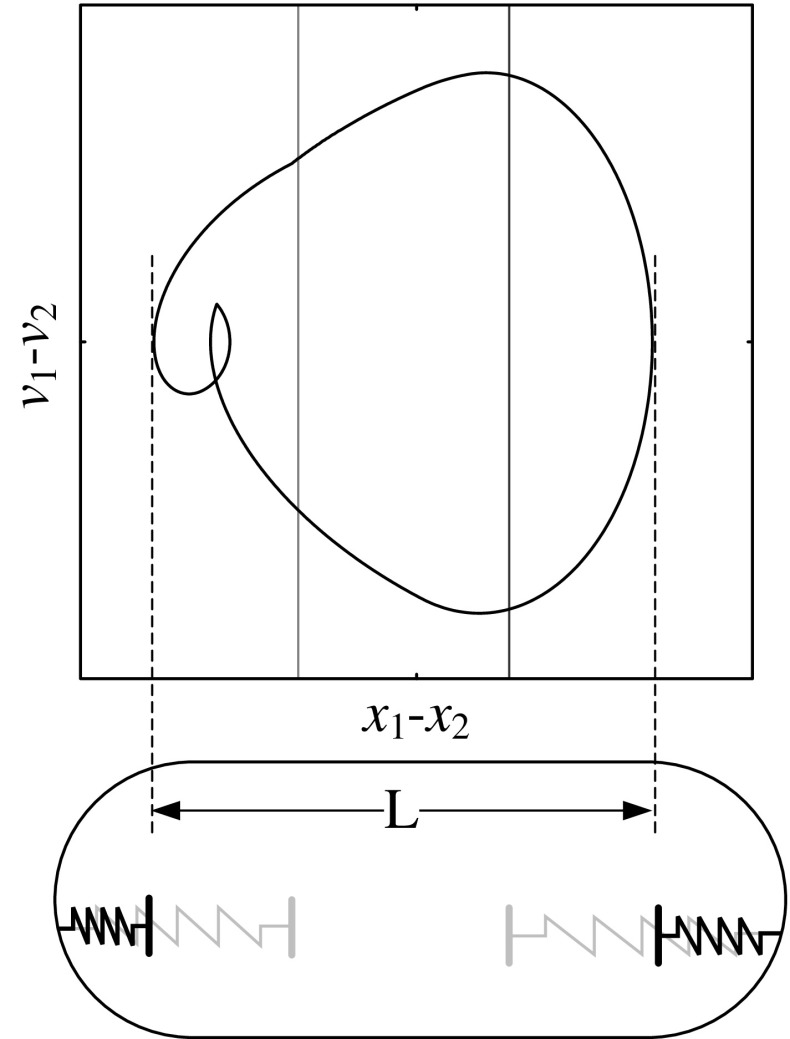
Required cabin length of the capsule which is determined by the relative displacement between the inner mass and the capsule, $x_1-x_2$. Locations of the left and the right impact surfaces are shown by *blue* and *red lines*, respectively. (Color figure online)

Another consideration of optimisation is space-saving for the capsule cabin, i.e. to use the smallest capsule to realise the fastest progression. Thus, our purpose is to shorten the cabin length of the capsule system as illustrated in Fig. 24, where the required cabin length is determined by the relative displacement between the inner mass and the capsule, $x_1-x_2$. Specifically, the shortest cabin length can be calculated as

14 $$\begin{aligned} L=\max (x_1-x_2)-\min (x_1-x_2), \end{aligned}$$

and we define the relative average progression velocity as

15 $$\begin{aligned} v_\text {L}=\frac{v_\text {avg}}{L}. \end{aligned}$$

**Fig. 25:**
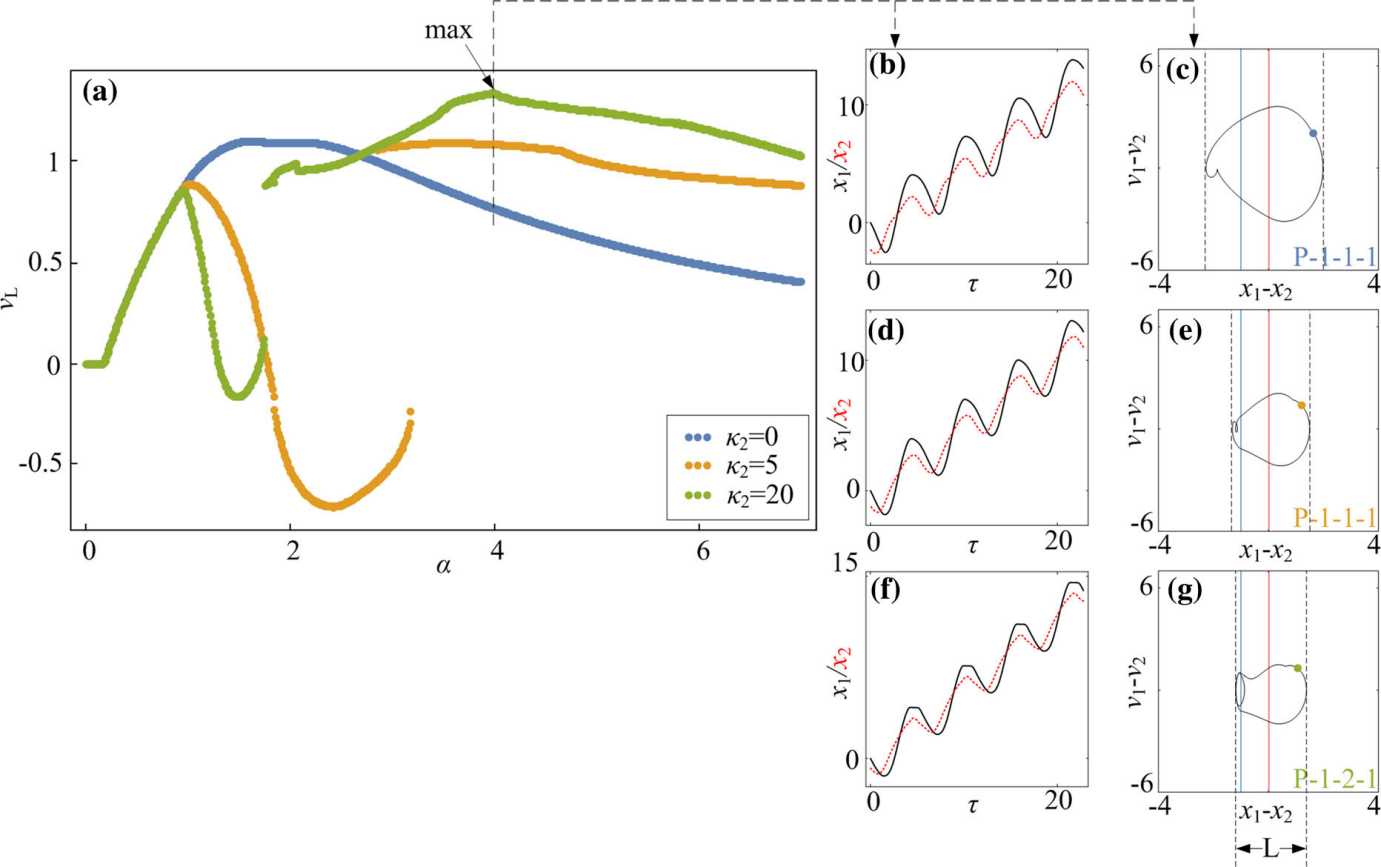
Relative average progression velocities with $\kappa _2=0$ (*blue dots*), $\kappa _2=5$ (*orange dots*), and $\kappa _2=20$ (*green dots*) are plotted as functions of amplitude of excitation $\alpha$ calculated for $\omega =1.1, \zeta =0.05, \gamma =1, \delta _1=0.02, \delta _2=1.0$, and $\kappa _1=2$. Additional windows demonstrate the time histories of displacements of the inner mass (*black line*) and the capsule (*red line*) and the trajectories on the phase plane ($x_1-x_2, v_1-v_2$) obtained for $\alpha =3.98$, **b**, **c** $\kappa _2=0$, **d**, **e** $\kappa _2=5$, and **f**, **g** $\kappa _2=20$, where the minimal requirement of cabin length $v_\text {L}$ is recorded. Locations of the left and the right impact surfaces are shown by *blue and red lines*, respectively. Poincaré sections are marked by blue, orange, and green dots on the phase plane. (Color figure online)

The calculations of the relative average progression velocity were carried out, and the results are presented in Fig. 25. As can be seen from the figure, the optimum progression for the minimal requirement of cabin length is obtained at $\alpha =3.98$, where a P-1-2-1 motion is recorded. Comparing the capsules with different left springs, the system with a strong left spring ($\kappa _2=20$) has the largest velocity and the minimal requirement of cabin length as demonstrated in Fig. 25f, g.

## Concluding remarks

Vibro-impact dynamics of the capsule systems with one-sided and two-sided constraints were studied in this paper. Our concern focused on optimising the control parameters of these system, i.e. mass ratio, stiffness ratios, gaps of contact, frequency and amplitude of excitation, in terms of average progression velocity, energy consumption, and cabin length. Bifurcation analysis was conducted by monitoring the relative velocity between the inner mass and the capsule and the average progression of the capsule per period of external excitation. Extensive comparative studies were undertaken for three different capsule systems, i.e. the capsule with a right constraint, the capsule with a right and a weak left constraints, and the capsule with a right and a strong left constraints. Our bifurcation studies revealed that the behaviour of the capsule with one-sided constraint was mainly periodic, and its average velocity was always faster than the capsule with two-sided constraints. The dynamic responses of the capsule with two-sided constraints may become very complex when the stiffness of the left spring increases.

For the investigation of influence of mass ratio $\gamma$, it was found that for the considered set of parameters, the system experienced period-1 motion for all the studied values of mass ratio, and the direction of capsule progression can be altered through the grazing contact between the inner mass and the right constraint. As the mass ratio increases, vibro-impact motion of the inner mass becomes ineffective so that average progression of the capsule decreases. For the scenario of the capsule with two-sided symmetrical constraints, i.e. $\delta _1=\delta _2$ and $\kappa _1=\kappa _2$, the capsule cannot progress for any values of the mass ratio. By investigating the influence of stiffness ratios $\kappa _1$ and $\kappa _2$, our studies indicated that the maximal average speed can be achieved by the capsule with one-sided constraint, and introduction of the second constraint with any values of stiffness may reduce the average speed of the capsule. Based on the bifurcation study of amplitude of excitation, it was found that the capsule with a right and a strong left constraints had more complex responses than the other two capsules. Furthermore, the capsule with a right and a weak left constraints could move faster than the others providing that a sufficiently large amplitude of excitation is applied. Our investigation on the frequency of excitation shows that, when the frequency is low ($\omega <0.5$), the dynamic responses of the capsule are complex, and any small perturbation on the frequency may results in a significant change of its average velocity. Once the frequency is sufficiently large ($\omega >1.5$), the dynamic responses of the system are mainly period-1 motions, and the grazing contact with the left constraint could help to enhance the progression speed of the capsule.

In addition, influence of gap of contact for the left constraint was studied. We have conducted the investigation for both positive and negative gaps, which the later one represents a prestressed internal mass. Our investigation suggests that if the miniaturization of the capsule is required, the prestressed constraint using a weak spring could be a viable option for prototype design, although the introduction of the left spring may affect the average speed of the capsule. We have also observed two important bifurcations which are the grazing bifurcation for the transition to backward drift and the boundary-intersection crossing bifurcation. They are important to be identified, since avoidance of such bifurcations could significantly reduce the energy loss caused by external friction so that improving energy efficiency of the entire capsule system.

Finally, calculations regarding to energy efficiency and cabin length were carried out. Our calculated results revealed that the capsule with a right constraint was the most energy-efficient, and the capsule with a right and a strong left constraints required the minimal cabin length. Based on the analyses above, our strategy for optimisation can be summarised as follows. When capsule speed is paramount, one can employ the two-sided capsule with a weak left constraint and apply a large amplitude of excitation. When energy consumption is taken into account, the one-sided capsule is preferable. When a miniaturized capsule prototype is needed, the two-sided capsule with a strong left constraint is the best choice for prototyping.

In conclusion, our motivation to investigate various design aspects of the vibro-impact capsule system for pipeline inspection was achieved by taking a comparative study on the capsule systems with one-sided and two-sided constraints. Optimum design parameters (e.g. stiffness ratios, mass ratio) and control parameters (e.g. frequency and amplitude of excitation) were suggested for prototype design with regards to capsule speed, energy efficiency, and capsule dimension, which are the main contribution of this paper. Future works will focus on implementation of the capsule prototype and its experimental testing in a fluid pipeline. It is also worth to develop a general control method for such type of nonlinear systems, e.g. [31, 32], particularly when the number of nonlinearities increases, the dynamics of the system will become more complex from periodic motion to chaos.
